# A null mutation of *C. elegans* *vwa-8*

**DOI:** 10.17912/micropub.biology.000263

**Published:** 2020-06-07

**Authors:** Ming Zhu, Andrew D Chisholm, Yishi Jin

**Affiliations:** 1 Section of Neurobiology, University of California San Diego, La Jolla, CA 92093, United States

**Figure 1:**
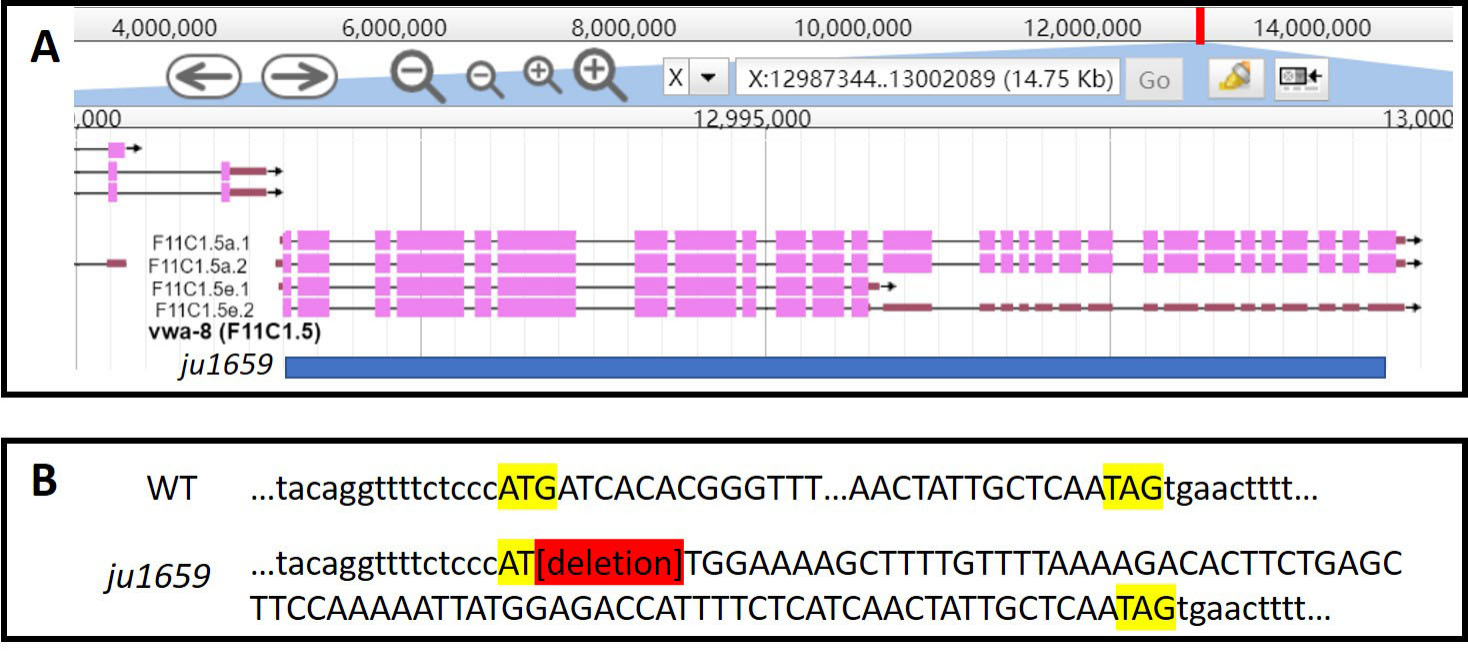
**(A)** shown is *vwa-8* gene structure from WormBase (Version: WS276). Blue bar indicates *vwa-8(ju1659)* deletion. **(B)** *vwa-8(ju1659)* deletes 7989bp. The start and stop codons of the F11C1.5a.1 isoform are highlighted in yellow. Exonal sequences are shown in uppercase.

## Description

VWA8 proteins, named for von Willebrand factor A (VWA) domain containing 8, are conserved from worm to mammals (Whittaker & Hynes, 2002). In human, two SNPs (rs9566845 and rs9566867) in vwa8 are found to be associated with bipolar disorder with comorbid migraine (Oedegaard *et al.*, 2010). Another SNP (rs9532931) is tentatively associated with a specific sub-group of autism patients (Anney *et al.*, 2010). The *C. elegans* VWA-8 long isoform shares 38% and 55% amino acid sequence identity and similarity, respectively, with human VWA8 long isoform. We showed that endogenous VWA-8 is expressed in mitochondria of somatic tissues, except neurons (Zhu, Chisholm & Jin, 2020). To determine the function of *C. elegans* *vwa-8*, we generated a null allele *vwa-8*(*ju1659*) by CRISPR-Cas9. *vwa-8*(*ju1659*) mutants are homozygous viable, and indistinguishable from wild type in gross phenotypes such as body size, brood size, growth rate and movement.

## Methods

We generated *vwa-8*(*ju1659*) using two CRISPR RNA (crRNAs), 5’-AGTGAAACCCGTGTGATCAT-3’ and 5’-CTACAACGAGAGTTGCCTGT-3’ (Integrated DNA Technologies) targeting the start and stop codons of *vwa-8*, respectively. The crRNAs were injected into wild type hermaphrodites with purified Cas9 protein (MacroLabs, University of California Berkeley), trans-activating crRNA (tracrRNA) and *dpy-10* crRNA, as described (Paix, Folkmann, Rasoloson, & Seydoux, 2015). The dumpy F1 progeny of the injected P0 wild type animals were singled to separate plates. F1 dumpy worms were then genotyped for the presence of potential *vwa-8* deletions using the following primers: 5’-CCTCGAGGGCCCCATATTTT-3’ and 5’-TGCTCTCGAACACCTTGCTT-3’. Several independent *vwa-8* deletions were identified. All deletion mutants behaved similarly. *ju1659* is a 7989bp deletion of *vwa-8* whichremoves nearly all the coding sequence of *vwa-8*, except the last 82bp of the last exon. CZ26606 *vwa-8*(*ju1659*) was generated after outcrossing with N2 for 3 times to remove the dumpy mutation.

## Reagents

CZ26606 *vwa-8*(*ju1659*) will be available at the CGC*.*
